# Morphological correlates of pyramidal cell axonal myelination in mouse and human neocortex

**DOI:** 10.1093/cercor/bhae147

**Published:** 2024-04-12

**Authors:** Maria Pascual-García, Maurits Unkel, Johan A Slotman, Anne Bolleboom, Bibi Bouwen, Adriaan B Houtsmuller, Clemens Dirven, Zhenyu Gao, Sara Hijazi, Steven A Kushner

**Affiliations:** Department of Psychiatry, Erasmus MC, Doctor Molewaterplein 40, Rotterdam, 3015 GD, The Netherlands; Department of Psychiatry, Erasmus MC, Doctor Molewaterplein 40, Rotterdam, 3015 GD, The Netherlands; Erasmus Optical Imaging Centre, Department of Pathology, Erasmus MC, Doctor Molewaterplein 40, Rotterdam, 3015 GD, The Netherlands; Department of Neuroscience, Erasmus MC, Doctor Molewaterplein 40, Rotterdam, 3015 GD, The Netherlands; Department of Neurosurgery, Erasmus MC, Doctor Molewaterplein 40, Rotterdam, 3015 GD, The Netherlands; Department of Neuroscience, Erasmus MC, Doctor Molewaterplein 40, Rotterdam, 3015 GD, The Netherlands; Department of Neurosurgery, Erasmus MC, Doctor Molewaterplein 40, Rotterdam, 3015 GD, The Netherlands; Erasmus Optical Imaging Centre, Department of Pathology, Erasmus MC, Doctor Molewaterplein 40, Rotterdam, 3015 GD, The Netherlands; Department of Neurosurgery, Erasmus MC, Doctor Molewaterplein 40, Rotterdam, 3015 GD, The Netherlands; Department of Neuroscience, Erasmus MC, Doctor Molewaterplein 40, Rotterdam, 3015 GD, The Netherlands; Department of Psychiatry, Erasmus MC, Doctor Molewaterplein 40, Rotterdam, 3015 GD, The Netherlands; Department of Pharmacology, University of Oxford, Mansfield Road, Oxford, OX1 3QT, United Kingdom; Department of Psychiatry, Erasmus MC, Doctor Molewaterplein 40, Rotterdam, 3015 GD, The Netherlands; Department of Psychiatry, Columbia University, 1051 Riverside Drive, New York, NY 10032, United States; SNF Center for Precision Psychiatry & Mental Health, Columbia University, 630 West 168th Street, New York, NY 10032, United States

**Keywords:** myelination, axonal morphology, human, mouse, cerebral cortex

## Abstract

The axons of neocortical pyramidal neurons are frequently myelinated. Heterogeneity in the topography of axonal myelination in the cerebral cortex has been attributed to a combination of electrophysiological activity, axonal morphology, and neuronal–glial interactions. Previously, we showed that axonal segment length and caliber are critical local determinants of fast-spiking interneuron myelination. However, the factors that determine the myelination of individual axonal segments along neocortical pyramidal neurons remain largely unexplored. Here, we used structured illumination microscopy to examine the extent to which axonal morphology is predictive of the topography of myelination along neocortical pyramidal neurons. We identified critical thresholds for axonal caliber and interbranch distance that are necessary, but not sufficient, for myelination of pyramidal cell axons in mouse primary somatosensory cortex (S1). Specifically, we found that pyramidal neuron axonal segments with a caliber < 0.24 μm or interbranch distance < 18.10 μm are rarely myelinated. Moreover, we further confirmed that these findings in mice are similar for human neocortical pyramidal cell myelination (caliber < 0.25 μm, interbranch distance < 19.00 μm), suggesting that this mechanism is evolutionarily conserved. Taken together, our findings suggest that axonal morphology is a critical correlate of the topography and cell-type specificity of neocortical myelination.

## Introduction

Axonal myelination is a crucial mammalian neurobiological adaptation that functions as an electrical insulator, enabling saltatory conduction of action potentials (Nave and Werner 2014), facilitating reliably timed neuronal activity (Salami et al. 2003; Ford et al. 2015), and optimizing neuronal energy expenditure (Wang et al. 2008). Accordingly, loss or damage to the myelin sheath or oligodendrocyte integrity has been shown to contribute to a variety of neuropsychiatric disorders (Snaidero et al. 2014). Developmentally, central nervous system (CNS) myelination exhibits protracted maturation throughout childhood, adolescence, and early adulthood, with considerable cell-type heterogeneity, as well as across distinct subcortical layers and brain areas (Brody et al. 1987; Kinney et al. 1988). In particular, the topography of myelination along individual axons of neocortical pyramidal neurons is known to be highly heterogeneous (Tomassy et al. 2014; Stadelmann et al. 2019; Call and Bergles 2021). This has raised the question of how oligodendrocytes determine their targets among the totality of axons. Intrinsic molecular cues from axons have been suggested to inhibit or attract myelinating oligodendrocytes (Redmond et al. 2016; Mount and Monje 2017). Moreover, neuronal activity has been shown to modulate oligodendrogenesis and axonal ensheathment (Nagy et al. 2017). However, although neocortical pyramidal cell axons are a well-characterized target of myelinating oligodendrocytes, the heterogeneity of their internodal topography remains poorly understood.

Axonal diameter is widely known to be an important determinant of myelination. Schwann cells, the myelinating cells in the peripheral nervous system, almost exclusively ensheath axons with a diameter greater than ~1 μm (Duncan 1934; Voyvodic 1989; Fraher and Dockery 1998). In the CNS, oligodendrocytes have been reported to restrict their ensheathment to axons with a diameter greater than ~0.3 μm (Lee et al. 2012; Stedehouder et al. 2019; Call and Bergles 2021). However, many axons exceeding this minimum threshold remain unmyelinated (Stedehouder et al. 2019). This suggests that in the CNS, axonal myelination is regulated by additional determinants. Previous studies of local fast-spiking interneurons in the cerebral cortex demonstrated that axonal morphology, including caliber and interbranch segment length, is sufficient to predict interneuron myelination with high accuracy (Stedehouder et al. 2019). Whether pyramidal cells adhere to similar or distinct rules governing their myelination has yet to be determined.

Here, we investigated the relationship between pyramidal cell axonal morphology and myelination in layer II/III of mouse primary somatosensory cortex (S1). We observed that in contrast to fast-spiking interneurons, axonal caliber and segment length are necessary but not sufficient for neocortical pyramidal cell segmental myelination. Lastly, using human ex vivo neurosurgically resected tissue, we found that the critical morphological thresholds necessary for neocortical pyramidal cell myelination in mice also extend to humans. Taken together, local axonal morphology of pyramidal cells appears to explain a substantial proportion of the variance underlying segmental myelination.

## Materials and methods

### Mice

All experiments were conducted under the approval of the Dutch Ethical Committee and in accordance with the Institutional Animal Care and Use Committee (IACUC) guidelines. *C57BL/6J* mice (referred as wild-type (WT); strain #000664) from both sexes between 8 and 12 weeks old were used in these experiments. Mice were group housed and maintained on a regular 12 h light/dark cycle at 22°C (±2°C) with ad libitum access to food and water.

### Human

Peri-tumoral infiltrated neocortical tissue was obtained from 5 patients undergoing tumor resection surgery at the Department of Neurosurgery (Erasmus University Medical Center, Rotterdam, The Netherlands). All procedures regarding human tissue were performed with the approval of the Medical Ethical Committee of the Erasmus University Medical Center. Written informed consent was provided by each participant in accordance with the Helsinki Declaration.

- Patient 1 was a 52-year-old male with a tumor in the right temporal lobe secondary to a melanoma. There were no episodes of epilepsy or mental illness associated. The patient did not receive antiepileptic medication.
- Patient 2 was a 62-year-old male with a glioblastoma on the right temporal lobe. The patient presented epilepsy secondary to the tumor.
- Patient 3 was a 32-year-old male with and oligodendroglioma on the right fronto-temporal lobe. The patient received treatment for epileptic seizures.
- Patient 4 was a 60-year-old male who underwent surgery due to a tumor in the right frontal lobe derived from a lung carcinoma. There was no past psychiatric history or presence of epilepsy or seizures.
- Patient 5 was a 69-year-old female who showed signs of glioblastoma in the right temporo-parietal lobe. There was no history of mental illness or presence of epilepsy.

### Electrophysiology

#### Mice

Mice were anesthetized using 5% isoflurane. After decapitation, brains were removed in ice-cold partial sucrose-based solution containing (in mM): sucrose 70, NaCl 70, NaHCO_3_ 25, KCl 2.5, NaH_2_PO_4_ 1.25, CaCl_2_ 1, MgSO_4_ 5, sodium ascorbate 1, sodium pyruvate 3, and D(+)-glucose 25 (carboxygenated with 5% CO_2_/95% O_2_). Coronal slices from the prefrontal and somatosensory cortex (300 μm thick) were obtained with a vibrating slicer (Microm HM 650V, Thermo Scientific) and incubated for 45 min at 34°C in holding artificial cerebrospinal fluid (ACSF) containing (in mM): 127 NaCl, 25 NaHCO_3_, 25 D(+)-glucose, 2.5 KCl, 1.25 NaH_2_PO_4_, 1.5 MgSO_4_, 1.6 CaCl_2_, 3 sodium pyruvate, 1 sodium ascorbate, and 1 MgCl_2_ (carboxygenated with 5% CO_2_/95% O_2_). Next, the slices recovered at room temperature for another 15 min.

Slices were then transferred into the recording chamber where they were continuously perfused with recording ACSF (in mM): 127 NaCl, 25 NaHCO_3_, 25 D-glucose, 2.5 KCl, 1.25 NaH_2_PO_4_, 1.5 MgSO_4_, and 1.6 CaCl_2_. Cells were visualized using an upright microscope (BX51WI, Olympus Nederland) equipped with oblique illumination optics (WI-OBCD; numerical aperture 0.8) and a 40× water-immersion objective. Images were collected by a CCD camera (CoolSMAP EZ, Photometrics) regulated by Prairie View Imaging software (Bruker). Layer II-III pyramidal cells in the somatosensory cortex were identifiable by their location and morphology.

Electrophysiological recordings were acquired using HEKA EPC10 quattro amplifiers and Patchmaster software (10 Hz sampling rate) at 33°C. Patch pipettes were pulled from borosilicate glass (Warner instruments) with an open tip of 3.5–5 M□ of resistance and filled with intracellular solution containing (in mM) 125 K-gluconate, 10 NaCl, 2 Mg-ATP, 0.2 EGTA, 0.3 Na-GTP, 10 HEPES and 10 K2-phosphocreatine, pH 7.4, adjusted with KOH (280 mOsmol/kg), with 5 mg/mL biocytin to fill the cells. Series resistance was kept under 20 M with correct bridge balance and capacitance fully compensated; cells that exceeded this value were not included in the study. Cells were filled with biocytin for at least 20 min.

Data analysis was performed offline using AxoGraph X Office software (v1.7.0, AxoGraph Scientific). Physiological characteristics were determined from voltage responses to current injection pulses of 500 ms duration in 100 pA intervals ranging from −100 to +600. Action potential (AP) characteristics were obtained by the first elicited AP in the voltage response; AP peak was defined as the maximum peak of the AP; AP half-width was measured as the half of the peak amplitude. AP threshold was determined as the first inflection point where the rising membrane potential exceeded 50 mV/ms slope. AP rise time was quantified as duration from 10 to 90% of the peak amplitude. AP decay time was measured as the duration from 100 to 50% of the peak. The afterhyperpolarization amplitude was measured as the hyperpolarizing peak from the initiation of the refractory period until the recovery state. AP frequency was estimated by the inverse of the difference between 2 consecutive AP in each current step. Likewise, passive membrane properties such as input resistance or conductance were calculated by the slope of the linear regression through the voltage–current curve. Excitatory postsynaptic currents (EPSC) were recorded at −70 mV holding potential during 5 min. Synaptic events were analyzed using MiniAnalysis Program (Synaptosoft, Decatur, Georgia).

#### Human

Ex vivo human recordings of acutely resected cortex slices were obtained by overlying tissue that was removed to gain access to the tumor. After resection, the tissue block was transferred into carboxygenated (95% O_2_/5% CO_2_) ice-cold solution and sliced into 300 μm thick slices for electrophysiology. For the electrophysiological recordings, only slices where there was no infiltrating tumor found were utilized. Whole-cell recordings and data analysis were performed and analyzed identically as the mouse tissue, except for current injection pulses that range from −100 to +600 in 50 pA steps.

### Immunohistochemistry

#### Mice biotin-filled cells

Layer II-III pyramidal neurons were filled with 5 mg/mL biocytin during whole-cell recordings and then fixed with 4% (PFA) overnight and stored in PBS at 4°C. Slices were rinsed with PBS and then stained with streptavidin-Cy3 secondary antibody (1:300; Invitrogen), 0.4% Triton X-100 and 2% NHS in PBS during 3 h. Slices were mounted on slides and coverslipped with 150 μL Mowiol (Sigma). After imaging with confocal at 63×, cells were unsealed and rinsed with PBS. To prevent thinning and dehydration, the slices were left in 30% sucrose overnight before resectioning. Coronal sections (40 μm thick) were recut using a freezing microtome (Leica, Wetzlar, Germany; SM 2000R) and stored in 0.1 M PB. Sections were blocked with 0.5% Triton X-100% (MerkMillipore) and 10% normal horse serum (NHS; Invitrogen, Bleiswijk, The Netherlands) for 1 h at room temperature, and incubated over 72 h at 4°C with mouse anti- myelin basic protein (MBP) (1:300, Santa Cruz, F-6, sc-271524), in PBS buffer containing 0.4% Triton X-100 and 2% NHS. MBP was visualized using anti-mouse Alexa488 secondary antibody (1:300, Invitrogen). Secondary antibodies were incubated at room temperature for 2 h in a PBS buffer containing 0.4% Triton X-100 and 2% NHS. Sections were then washed with PBS and cover-slipped in Vectashield H1000 fluorescent mounting medium (Vector Labs, Peterborough, United Kingdom).

#### Human biocytin-filled cells

Human pyramidal cells were filled with 5 mg/mL biocytin and fixed in 4% PFA overnight. Slices were washed with PBS and stained with streptavidin-Cy3 secondary antibody (1:300, Invitrogen) in a PBS-based solution containing 0.4% Triton X-100 and 5% bovine serum albumin (BSA; Sigma-Aldrich, The Netherlands) during 3 h at room temperature. Slices were mounted and coverslipped. Slices were recut in 40 μm slices and then blocked in PBS containing 0.4% Triton X-100 and 5% BSA and posteriorly stained using mouse anti-MBP (1:300, Santa Cruz, F-6, sc-271524) in PBS, 0.4% Triton X-100 and 5% BSA during 72 h at 4°C. Secondary antibodies Alexa-488 anti-mouse and streptavidin-Cy3 were diluted in PBS, 0.4% Triton X-100 and 5% BSA during 3 h at room temperature. Slices were then cover-slipped and sealed for imaging.

### Confocal imaging and reconstruction

Images were taken using a Zeiss LSM 700 microscope (Carl Zeiss) equipped with Plan-Apochromat 10×/0.45 NA, 40×/1.3 NA (oil immersion), and 63×/1.4 NA (oil immersion) objectives. Alexa488 and Cy3-secondary fluorophores were imaged using excitation wavelengths of 488 and 555 nm, respectively.

In WT and human cells, a 10× magnification picture of the cell was used to quantify the distance from soma to pia, and a whole-cell overview image was acquired using 63× magnification objective and 555 nm wavelength. Pinhole was kept at 0.2% and gain was set at 750–800 units to adjust the signal-to-noise ratio. Biocytin-filled cells were imaged with tiled *z*-stack images (512 × 512 pixels) with a step size of 1 μm. Resectioned slices of such cells were obtained at 40× magnification using 488 and 555 nm wavelength filters. MBP staining pictures were acquired using 40× magnification (1024 × 1024 pixels) and excitation wavelength of 555 nm. The same settings were maintained across all pictures to ensure fluorescence was equally measured.

Overview images were then transferred into Neurolucida 360 software (v2.8; MBF Bioscience) and the axon was reconstructed using the interactive tracing with the Directional Kernels method. Reconstruction of the axon and myelinated segments were analyzed with Neurolucida Explorer (MBF Bioscience). Distance to the pia and internodes were quantified and observed using ImageJ (ImageJ 5.12h). Axons were considered myelinated when they exhibited at least 1 MBP-positive internode, and unmyelinated when no MBP-positive internodes could be identified up to at least the 10th branch order. The distance to the first branch point was calculated as the length of the axon from the soma to the first branching axon segment. The distance to the first myelinated segment was determined as the distance along the axon from the soma to the initial point of the MBP segment.

### Structured illumination microscopy

Structured illumination imaging was performed using a Zeiss Elyra PS1 system (Carl Zeiss). 3D structured illumination microscopy (SIM) data were acquired using a 63×/1.4 NA oil immersion objective. A 561 nm 100 mW diode laser together with a BP 570–650 + LP 750 filter was used to excite the fluorophores. Five phases and 5 rotations modulated the grating that was present in the light path during 3D-SIM acquisition. Serial z-stacks of 110 nm were recorded on an Andor iXon DU 885, 1002 × 1004 EMCCD camera. Raw data were reconstructed using Zen 2012 software (Zeiss) and posteriorly analyzed in Fiji image analysis software.

To avoid overexposure from the soma, the first picture was taken starting from the second branch order axonal segment and continuing anterograde. Axonal segments were imaged from branch point to the subsequent branch point. Only axonal segments that were entirely within the microscopy image were taken into consideration. Bandwidth, offset, and exposure time were regulated manually per segment to prevent oversaturation of the picture. Reconstructed pictures were loaded into Fiji and analyzed using a custom-made script as previously described (Stedehouder et al. 2019).

The axonal diameter of each individual segment was quantified using the average intensity projection of each individual axonal segment, traced using Simple Neurite Tracer to mark the path along which serial 40 nm orthogonal sections of the axon were extracted along the entire segment length. For each successive orthogonal section, a Gaussian curve was fitted to the intensity profile. Only those fits with *r^2^* > 0.9 were included in the analysis. The full width at half maximum was calculated using the corresponding Gaussian curve and employed to calculate the axonal diameter for each orthogonal section. The average axonal diameter of each individual segment was then estimated and used for further analysis.

### Statistical analysis

All statistical analyses were operated using GraphPad 8.01. Data were firstly analyzed for normality using Kolmogorov–Smirnov test. No outlier was identified or removed. Data sets following normal distribution were analyzed using unpaired 2-tailed *t*-test. Data sets without a normal distribution were analyzed using Mann Whitney test.

A self-custom-made algorithm for R was created to calculate the receiver operating characteristic (ROC) curve, the area under the curve (AUC), and the thresholds from the pure length and diameter univariate models and for the bivariate model. The optimum thresholds were determined as the maximum point of the Youden’s J statistic, calculated as the sum of the sensitivity and specificity minus 1. The AUC was estimated as the integral of the ROC curves for the univariate and bivariate models.

## Results

### Myelination of pyramidal cells in mouse somatosensory cortex is associated with axonal morphology

**Fig. 1:**
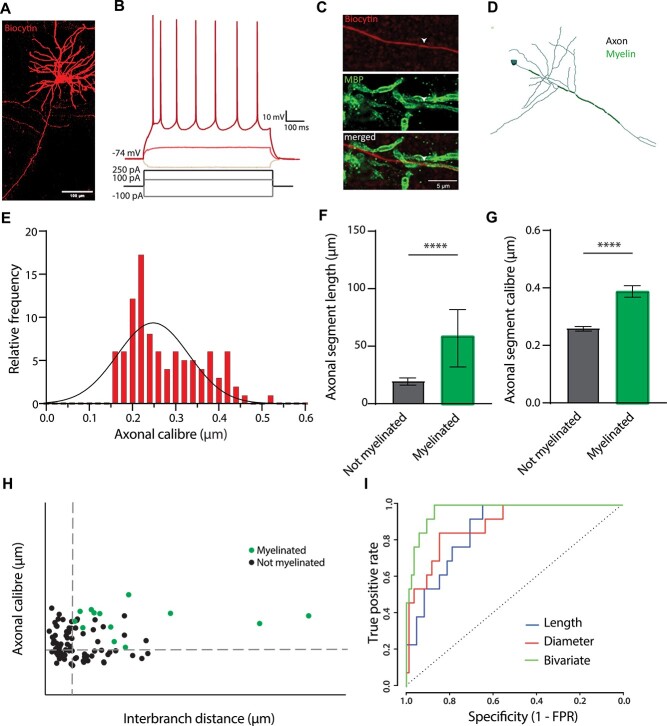
**Axonal properties of pyramidal cells in the mouse S1 layer II-III.** (**A**) Example of a pyramidal cell in S1 filled with biocytin (scale bar = 100 μm). (**B**) Electrophysiological example traces of a layer II-III pyramidal cell at hyperpolarizing (light red) and depolarizing currents (red and dark red). (**C**) High magnification picture of a myelinated segment (biocytin in upper panel; MBP in middle panel, merged picture in lower panel; scale bar = 5 μm). Arrow heads indicate the beginning of a myelinated axonal segment. (**D**) Neurolucida reconstruction of a representative S1 pyramidal cell including the myelinated segments in the main axon (axon in dark; myelin in green). (**E**) Frequency histogram of the axonal segment caliber, fitted with a Gaussian curve; mean = 0.28 μm ± 0.008 μm s.e.m.; *n* = 98 axonal segments from 10 cells. (**F**) Comparison of the axonal segment length between myelinated and unmyelinated segments (Mann–Whitney: U = 149; *P*-value < 0.0001; unmyelinated segments = 14.71 μm; myelinated segments = 43.56 μm; *n* = 98 segments). (**G**) Caliber of myelinated and unmyelinated segments (*t*-test: *t* = 5.851; *P*-value < 0.0001; unmyelinated segments = 0.26 μm; myelinated segments = 0.39 μm; *n* = 98 segments). (**H**) Distribution of the axonal segment caliber and interbranch distances for myelinated (green) and unmyelinated (black) segments. Dotted lines depict the bivariate thresholds indicated in figure. (**I**) ROC curves for univariate (length or caliber) and bivariate analyses.

SIM imaging of axonal morphology and myelination of pyramidal cells in the somatosensory cortex (S1) was performed following whole-cell patch-clamp recordings in S1 layer II-III from WT mice at 8–12 weeks of age (Fig. 1A and B; Supplementary Fig. 1; Supplementary Table 1a). Biocytin-labeled neurons were imaged using confocal microscopy and immunolabeled with MBP to investigate their myelination profile (Fig. 1C). A total of 80% (8 out of 10) of the examined cells were myelinated. Consistent with previous studies (Tomassy et al. 2014), only the primary axon had myelinated segments (Fig. 1D; Supplementary Table 1b). Axonal diameter and length of each reconstructed segment were measured using SIM imaging as described by Stedehouder et al. (2019). Axonal shaft diameter, independent of myelination status, had an average diameter of 0.26 μm (Fig. 1E). Importantly, segments that exhibited myelination had a consistently larger caliber compared with those that were unmyelinated. Moreover, myelinated segments were longer on average than those that were unmyelinated (Fig. 1F and G).

Using ROC analysis, we examined the sufficiency of axonal caliber and length to predict segmental myelination in S1 pyramidal neurons. As expected, axonal diameter was a moderate predictor of segmental myelination (threshold, 0.35 μm; AUC = 0.97; sensitivity = 0.85; specificity = 0.85). Axonal length was also significantly associated with segmental myelination, albeit with a somewhat lower specificity (threshold, 18.90 μm; AUC = 0.87; sensitivity = 1; specificity = 0.65). Therefore, we performed a bivariate ROC analysis as previously described (Stedehouder et al. 2019), to determine whether the joint combination of axonal caliber and length might generate better estimates of segmental myelination along pyramidal cell axons. The combination of both parameters yielded a significant yet mild improvement in the prediction of segmental myelination (AUC = 0.97; sensitivity = 1; specificity = 0.88), with critical thresholds for axonal caliber and length of 0.24 and 18.10 μm, respectively (Fig. 1H and I; axonal caliber: *P* < 0.01; axonal length: *P* < 0.01). A substantial proportion of the morphologically suprathreshold segments remained unmyelinated (58.3% myelinated, 14/24). In contrast, none of the segments below these morphological thresholds were myelinated (100% unmyelinated, 26/26). Together, these data suggest that the joint combination of axonal caliber > 0.23 μm and interbranch length > 18.10 μm is necessary, but not sufficient, for segmental myelination of S1 pyramidal neurons.

### Human neocortical pyramidal neurons exhibit a similar morphological relationship with axonal myelination

**Fig. 2:**
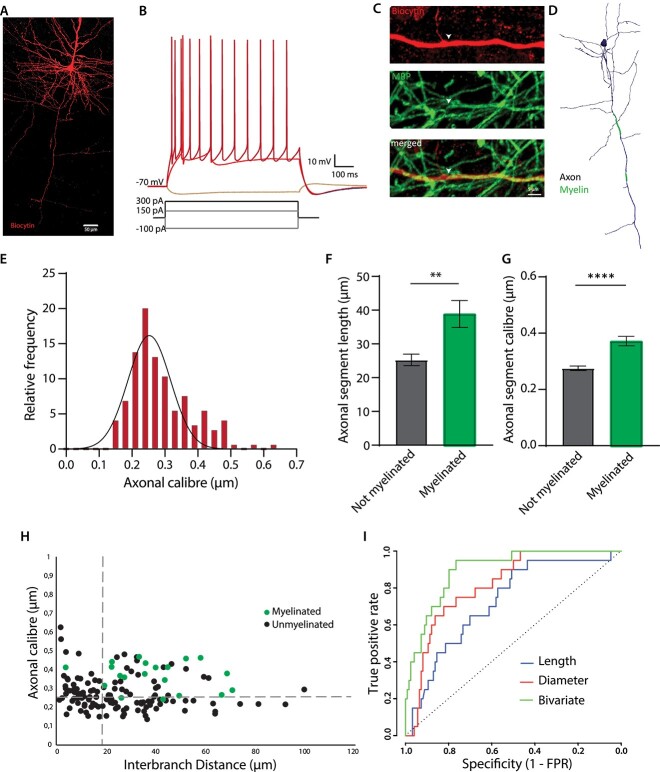
**Axonal properties of human pyramidal cells in layer II-III.** (**A**) Confocal image of a representative human pyramidal cell filled with biocytin (scale bar = 50 μm). (**B**) Electrophysiological example traces of a human pyramidal cell at hyperpolarizing (light red) and depolarizing currents (red [rheobase] and dark red). (**C**) High resolution image of a myelinated segment (MBP; middle panel) in a human pyramidal neuron (Biocytin; upper panel). Arrow heads indicate the beginning of a myelinated axonal segment (scale bar = 5 μm). (**D**) Neurolucida axonal reconstruction of a representative pyramidal cell (axon in dark; myelin in green). (**E**) Frequency histogram of the axonal segment caliber, fitted with a Gaussian curve; mean = 0.29 μm ± 0.008 μm s.e.m.; *n* = 144 axonal segments from 10 cells. (**F**) Axonal segments that were myelinated were significantly longer than those that did not present any internode ((*t*-test: *t* = 2.989; *P*-value = 0.003; unmyelinated segments: 25.28 μm; myelinated segments: 38.88 μm). (**G**) Axonal caliber of myelinated segments is larger than those unmyelinated (*t*-test: *t* = 4.602; *P*-value < 0.001; (unmyelinated segments: 0.27 μm; myelinated segments: 0.37 μm). (**H**) Distribution of the axonal segment caliber and interbranch distances for myelinated (green) and unmyelinated (black) segments. Dotted lines depict the critical thresholds of the bivariate analysis. (**I**) ROC curves for univariate (length and caliber) and bivariate analyses.

We next explored whether the thresholds observed in mouse pyramidal neurons also extend to human pyramidal neurons. To that end, we performed whole-cell electrophysiology and biocytin-filling of layer II/III human pyramidal cells in neurosurgically resected neocortical tissue (9 cells from 5 patients; see *Methods*). All recorded cells exhibited canonical electrophysiological characteristics of pyramidal neurons, including regular firing frequency, high amplitude, and subthreshold depolarization (Fig. 2A and B; Supplementary Table 1a). Post hoc MBP immunofluorescence revealed that 89% of the cells (8 out of 9) were myelinated with an average internode length of 49.37 ± 12.69 μm, total myelin length of 162.21 ± 82.30 μm, and 2.87 ± 1.25 number of internodes (Fig. 2C and D; Supplementary Table 1b).

SIM imaging of individual axonal segments yielded a mean axonal shaft diameter of 0.29 ± 0.77 μm (Fig. 2E). Similar to our observations in S1 pyramidal cells in mice, human neocortical pyramidal cells revealed a strong association between segmental myelination and the joint combination of axonal diameter and axonal length. Segments that lacked myelination exhibited shorter length and thinner diameters compared with those that were myelinated (Fig. 2F and G). ROC analysis of the univariate models revealed thresholds of 21.75 μm for segment length (AUC = 0.73; sensitivity = 0.90; specificity = 0.51) and 0.35 μm for axonal diameter (AUC = 0.82; sensitivity = 0.70; specificity = 0.82, respectively). The bivariate analysis that included the joint combination of axonal caliber and segment length revealed combined thresholds of 19.00 μm for segment length and 0.25 μm for axonal caliber (AUC = 0.90; sensitivity = 0.95; specificity = 0.77). The joint combination of both parameters significantly improved the ROC model compared with univariate models based on axonal caliber (*P* = 0.027) or segment length (*P* < 0.001) (Fig. 2H and I). Accordingly, 95.2% (20/21) of myelinated segments were above these critical bivariate thresholds.

The observed critical thresholds were highly similar in mouse (caliber, 0.24 μm; segment length, 18.10 μm) and human (caliber, 0.25 μm; segment length 19.00 μm). Myelination parameters for mouse and human cortical pyramidal neurons were similar, with the exception of distance to the first internode, which was longer in human pyramidal neurons (Supplementary Table 1b) (Beaulieu-Laroche et al. 2018; Poorthuis et al. 2018). Together, these data indicate that pyramidal cell myelination exhibits evolutionarily conserved morphological correlates in mouse and human neocortex.

## Discussion

Pyramidal cells are the major class of excitatory glutamatergic neurons in the cerebral cortex. Their function, morphology, excitability, and number vary across different layers of the neocortex. The myelination profile of pyramidal cells is highly diverse and depends on their electrophysiological activity and connectivity (Tomassy et al. 2014). However, few studies have previously been performed to investigate the cellular determinants underlying the spatial distribution of neocortical pyramidal cell internodes along individual axons. In the present study, we provide new insights regarding the relationship between axonal morphology and the myelination of individual segments along pyramidal cell axons. Our findings reveal a similar minimum necessary bivariate threshold for the myelination of human and mouse neocortical pyramidal cell axonal segments. These results could provide an explanation as to why typically only the primary axon is myelinated in neocortical pyramidal cells, as collateral branches protruding from the main axon of pyramidal neurons have a notably thinner diameter, which is frequently subthreshold for myelination of these segments.

Extrinsic and intrinsic cues guide oligodendrocyte precursor cells (OPCs) to mature into myelinating oligodendrocytes and ensheath axons (Zuchero and Barres 2013). Beyond fiber diameter, axonal curvature has also been suggested to play an important permissive role in myelination (Almeida 2018). Bechler et al. demonstrated that oligodendrocytes increase the sheath length in larger fibers (Bechler et al. 2015). Our results are consistent with a model whereby interbranch distance creates a physical constraint for initiation of myelination, given that segments shorter than 18 μm are rarely myelinated. Furthermore, axonal branch points are consistently unmyelinated, which might suggest that the angle formed by the emerging daughter branches biophysically restrict and/or molecularly inhibit oligodendrocyte ensheathment. Consistent with this finding, branch points of myelinated axons are also common locations for nodes of Ranvier and therefore critical for signal integration along the axon (Lei and Chiu 2001; Kole 2011).

Our findings revealed similar myelination thresholds in humans (18.96 μm for segment length and 0.25 μm for axonal diameter) and mice (18.14 and 0.24 μm, respectively), suggesting that oligodendrocytes might have intrinsic mechanisms for myelination that are highly preserved across species. Likewise, fast-spiking interneurons exhibit similar morphological thresholds (Stedehouder et al. 2019) as pyramidal neurons, suggesting that oligodendrocyte ensheathment potential might be independent of neuronal cell type and strongly determined by biophysical constraints. The only significant difference between species that we observed in the myelination parameters was the distance to first internode, which is likely due to the longer average length of pyramidal cell axons in human versus mouse neocortex. It is important to note that we have used SIM to quantify these morphological thresholds, which we acknowledge has lower spatial resolution (100–150 nm) compared with other techniques that have previously been used for imaging axonal myelination such as array tomography or electron microscopy. However, we have previously demonstrated the validity of our approach for accurately quantifying axonal myelination in the cerebral cortex (Stedehouder et al. 2019). Moreover, the use of SIM also facilitated the integration of electrophysiological and morphological measures at the level of individual neurons.

Another crucial observation resulting from this data is that in contrast to fast-spiking interneurons in which the observed bivariate axonal morphological thresholds were both necessary and sufficient for segmental myelination, it appears that while necessary, these morphological correlates are insufficient to explain a high proportion of the variance of axonal myelination along neocortical pyramidal neurons. Future studies will be required to quantitatively establish the full repertoire of determinants of pyramidal cell myelination. However, based on current understanding, this is also likely to include (i) neuronal activity, which has been shown to significantly alter myelination (Gibson et al. 2014), (ii) synaptic connectivity (Hughes and Appel 2019), (iii) differences across layers and brain regions (Tomassy et al. 2014), and (iv) molecular cues, such as chemoattractants and/or chemorepellents, which are known to influence myelination of axons by oligodendrocytes (Redmond et al. 2016).

Early neuron–oligodendrocyte interactions have been shown to play a role in developmental myelination of local interneurons (Mazuir et al. 2021). Previous studies have indicated that OPCs receive transient synaptic input from local PV interneurons and genetic inactivation of these neuro-glial synapses at an early stage of postnatal development leads to significant changes in interneuron myelination and maturation (Benamer et al. 2020). It has yet to be determined whether early neuron–oligodendrocyte interactions play similar roles in neocortical pyramidal neuron myelination. Moreover, in contrast to interneurons, the axonal projections of pyramidal cells are often not restricted locally but extend to other brain regions, highlighting potential differential functional implications for myelination between local interneurons and pyramidal neurons. Previous studies in mouse somatosensory cortex revealed that axons of deep layer (V/VI) pyramidal neurons have greater average myelin coverage and a distinct pattern of myelination topography compared with superficial layer (II/III) pyramidal neurons (Tomassy et al. 2014), highlighting the complexity of regional and cell-type specific mechanisms. Further studies will be required to elucidate whether the myelination thresholds observed here extend to pyramidal neurons in other layers and regions of the cerebral cortex.

In conclusion, our data suggest that pyramidal cell axonal morphology is a major contributor to myelin topography. Moreover, the predictive thresholds for axonal caliber and length are similar in mouse and human, highlighting the possibility of an evolutionarily conserved biophysical mechanism governing axonal myelination in the neocortex.

## Supplementary Material

Supplementary_data_bhae147

## References

[ref1] Almeida RG . The rules of attraction in central nervous system myelination. Front Cell Neurosci. 2018:12:367. 10.3389/fncel.2018.00367. 30374292 PMC6196289

[ref2] Beaulieu-Laroche L , Toloza EHS, van der Goes MS, Lafourcade M, Barnagian D, Williams ZM, Eskandar EN, Frosch MP, Cash SS, Harnett MT. Enhanced dendritic compartmentalization in human cortical neurons. Cell. 2018:175(3):643–651.e14. 10.1016/j.cell.2018.08.045. 30340039 PMC6197488

[ref3] Bechler ME , Byrne L, French-Constant C. CNS myelin sheath lengths are an intrinsic property of oligodendrocytes. Curr Biol. 2015:25(18):2411–2416. 10.1016/j.cub.2015.07.056. 26320951 PMC4580335

[ref4] Benamer N , Vidal M, Balia M, Angulo MC. Myelination of parvalbumin interneurons shapes the function of cortical sensory inhibitory circuits. Nat Commun. 2020:11:5151. 10.1038/s41467-020-18984-7. 33051462 PMC7555533

[ref5] Brody BA , Kinney HC, Kloman AS, Gilles FH. Sequence of central nervous system myelination in human infancy. I. An autopsy study of myelination. J Neuropathol Exp Neurol. 1987:46(3):283–301. 10.1097/00005072-198705000-00005. 3559630

[ref6] Call CL , Bergles DE. Cortical neurons exhibit diverse myelination patterns that scale between mouse brain regions and regenerate after demyelination. Nat Commun. 2021:12(1):4767. 10.1038/s41467-021-25035-2. 34362912 PMC8346564

[ref7] Duncan D . The importance of diameter as a factor in myelination. Science. 1934:79(2051):363–363. 10.1126/science.79.2051.363. 17741794

[ref8] Ford MC , Alexandrova O, Cossell L, Stange-Marten A, Sinclair J, Kopp-Scheinpflug C, Pecka M, Attwell D, Grothe B. Tuning of Ranvier node and internode properties in myelinated axons to adjust action potential timing. Nat Commun. 2015:6(1):8073. 10.1038/ncomms9073. 26305015 PMC4560803

[ref9] Fraher J , Dockery P. A strong myelin thickness-axon size correlation emerges in developing nerves despite independent growth of both parameters. J Anat. 1998:193:195–201. 10.1046/j.1469-7580.1998.19320195.x. 9827635 PMC1467839

[ref10] Gibson EM , Purger D, Mount CW, Goldstein AK, Lin GL, Wood LS, Inema I, Miller SE, Zuchero JB, Barres BA, et al. Neuronal activity promotes oligodendrogenesis and adaptive myelination in the mammalian brain. Science. 2014:2:344(6183):125304. 10.1126/science.1252304. PMC4096908 24727982

[ref11] Hughes AN , Appel B. Oligodendrocytes express synaptic proteins that modulate myelin sheath formation. Nat Commun. 2019:10(1):4125. 10.1126/science.1252304. 31511515 PMC6739339

[ref12] Kinney HC , Brody BA, Kloman AS, Gilles FH. Sequence of central nervous system myelination in human infancy. II. Patterns of myelination in autopsied infants. J Neuropathol Exp Neurol. 1998:47(3):217–234. 10.1097/00005072-198805000-00003. 3367155

[ref13] Kole MHP . First node of Ranvier facilitates high-frequency burst encoding. Neuron. 2011:71(4):671–682. 10.1016/j.neuron.2011.06.024. 21867883

[ref14] Lee S , Leach MK, Redmond SA, Chong SYC, Mellon SH, Tuck SJ, Feng ZQ, Corey JM, Chan JR. A culture system to study oligodendrocyte myelination processes using engineered nanofibers. Nat Methods. 2012:9(9):917–922. 10.1038/nmeth.2105. 22796663 PMC3433633

[ref15] Lei Z , Chiu SY. Computer model for action potential propagation through branch point in myelinated nerves. J Neurophysiol. 2001:85(1):197–210. 10.1152/jn.2001.85.1.197. 11152720

[ref16] Mazuir E , Fricker D, Sol-Foulon N. Neuron–oligodendrocyte communication in myelination of cortical GABAergic cells. Life. 2021:11(3):216. 10.3390/life11030216. 33803153 PMC7999565

[ref17] Mount CW , Monje M. Wrapped to adapt: experience-dependent myelination. Neuron. 2017:95(4):743–756. 10.1016/j.neuron.2017.07.009. 28817797 PMC5667660

[ref18] Nagy B , Hovhannisyan A, Barzan R, Chen TJ, Kukley M. Different patterns of neuronal activity trigger distinct responses of oligodendrocyte precursor cells in the corpus callosum. PLoS Biol. 2017:15(8):e2001993. 10.1371/journal.pbio.2001993. 28829781 PMC5567905

[ref19] Nave KA , Werner HB. Myelination of the nervous system: mechanisms and functions. Annu Rev Cell Dev Biol. 2014:30(1):503–533. 10.1146/annurev-cellbio-100913-013101. 25288117

[ref20] Poorthuis RB , Muhammad K, Wang M, Verhoog MB, Junek S, Wrana A, Mansvelder HD, Letzkus JJ. Rapid neuromodulation of layer 1 interneurons in human neocortex. Cell Rep. 2018:23:951–958. 10.1016/j.celrep.2018.03.111. 29694902 PMC5946807

[ref21] Redmond SA , Mei F, Eshed-Eisenbach Y, Osso LA, Leshkowitz D, Shen YAA, Kay JN, Aurrand-Lions M, Lyons DA, Peles E, et al. Somatodendritic expression of JAM2 inhibits oligodendrocyte myelination. Neuron. 2016:91(4):824–836. 10.1016/j.neuron.2016.07.021. 27499083 PMC4990461

[ref22] Salami M , Itami C, Tsumoto T, Kimura F. Change of conduction velocity by regional myelination yields constant latency irrespective of distance between thalamus and cortex. Proc Natl Acad Sci USA. 2003:100(10):6174–6179. 10.1073/pnas.0937380100. 12719546 PMC156345

[ref23] Snaidero N , Möbius W, Czopka T, Hekking LHP, Mathisen C, Verkleij D, Goebbels S, Edgar J, Merkler D, Lyons DA, et al. Myelin membrane wrapping of CNS axons by PI(3,4,5)P3-dependent polarized growth at the inner tongue. Cell. 2014:156(1–2):277–290. 10.1016/j.cell.2013.11.044. 24439382 PMC4862569

[ref24] Stadelmann C , Timmler S, Barrantes-Freer A, Simons M. Myelin in the central nervous system: structure, function, and pathology. Physiol Rev. 2019:99:1381–1431. 10.1152/physrev.00031.2018. 31066630

[ref25] Stedehouder J , Brizee D, Slotman JA, Pascual-Garcia M, Leyrer ML, Bouwen BL, Dirven CM, Gao Z, Berson DM, Houtsmuller AB, et al. Local axonal morphology guides the topography of interneuron myelination in mouse and human neocortex. Elife. 2019:8:e48615. 10.7554/eLife.48615. PMC6927753 31742557

[ref26] Tomassy GS , Berger D, Chen HH, Kasthuri N, Hayworth K, Vercelli A, Seung S, Lichtman J, Arlotta P. Distinct profiles of myelin distribution. Science. 2014:2014(344):319–324. 10.1126/science.1249766. PMC4122120 24744380

[ref27] Voyvodic JT . Target size regulates calibre and myelination of sympathetic axons. Nature. 1989:342(6248):430–433. 10.1038/342430a0. 2586612

[ref28] Wang SSH , Shultz JR, Burish MJ, Harrison KH, Hof PR, Towns LC, Wagers MW, Wyatt KD. Functional trade-offs in white matter axonal scaling. J Neurosci. 2008:28(15):4047–4056. 10.1523/JNEUROSCI.5559-05.2008. 18400904 PMC2779774

[ref29] Zuchero JB , Barres BA. Intrinsic and extrinsic control of oligodendrocyte development. Curr Opin Neurobiol. 2013:23(6):914–920. 10.1016/j.conb.2013.06.005. 23831087 PMC4431975

