# Supplementary material for: Morphological correlates of pyramidal cell axonal myelination in mouse and human neocortex
Source: Cereb Cortex. 2024 Apr 12;34(4):bhae147. doi: 10.1093/cercor/bhae147 (PMC11014882; doi:10.1093/cercor/bhae147)
Supplement: Supplementary_data_bhae147 [file supplementary_data_bhae147.pdf]

Supplementary data

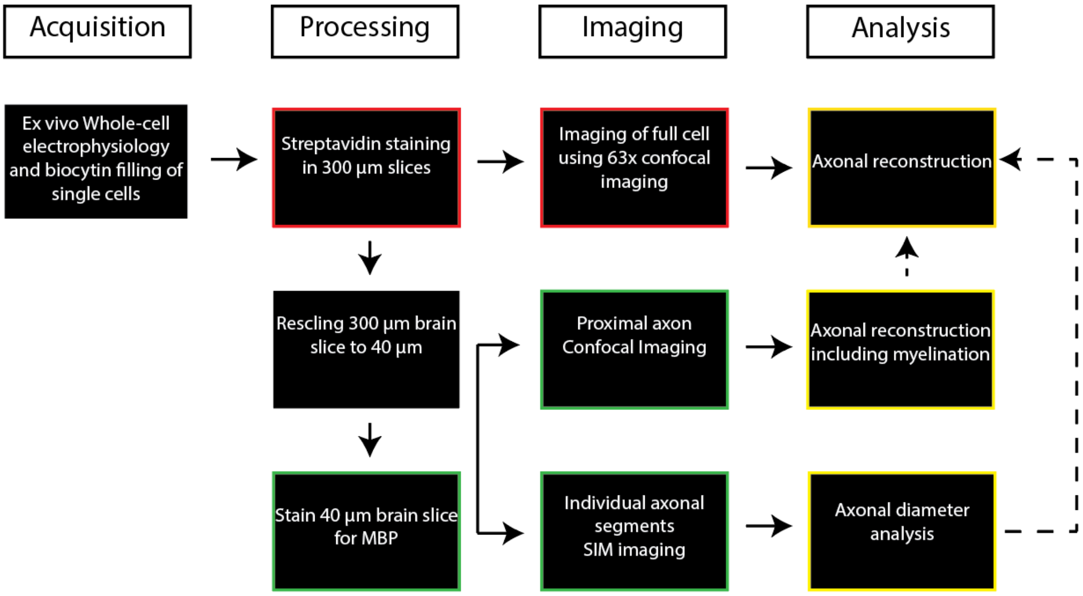

Supplementary Figure 1. Experimental design for axonal reconstructions.

	Mouse		Human		p value
**a)**	**Mean (Mouse)**	**SEM**	**Mean (Human)**	**SEM**	**t test or Fisher exact**
**Capacitance (pF)**	79.00	16.12	78.99	13.35	0.999
**RMP (mV)**	-74.09	1.86	-68.53	2.11	0.066
**Rinput (MΩ)**	115.23	19.08	189.14	34.67	0.066
**AP threshold (mV)**	-32.80	2.43	-40.79	3.25	0.062
**AP amplitude (mV)**	32.92	1.91	41.29	2.32	0.012
**AP peak (mV)**	89.86	2.98	94.00	3.14	0.356
**AP half-width (ms)**	1.12	0.13	0.96	0.03	0.268
**AP rise-time (ms)**	0.23	0.03	0.24	0.01	0.587
**AP decay time (ms)**	0.80	0.13	0.67	0.10	0.451
**Rheobase (pA)**	244.00	42.27	95.00	19.91	0.007
**b)**					
**Myelinated (ratio)**	0.80	0.13	0.88	0.13	0.657
**Myelin in first segment (ratio)**	0.50	0.19	0.38	0.18	0.934
**Number of internodes**	2.56	0.69	2.88	1.25	0.159
**Internode length (µm)**	22.96	3.45	41.31	17.68	0.345
**Total amount myelin (µm)**	83.85	23.65	162.21	82.30	0.391
**Distance to first internode (µm)**	54.64	16.28	149.68	27.14	0.011
**Average internode length (µm)**	22.96	3.45	41.31	17.68	0.345
**Distance to first branch point (µm)**	75.27	4.14	129.71	64.58	0.428
**Soma surface area (µm2)**	415.36	48.89	540.99	78.70	0.209

Supplementary Table 1. Electrophysiological properties and myelin profile of layer II-III pyramidal cells in human (n=8 cells) and mice (n=10 cells)**. a)** Intrinsic properties of LII-III pyramidal cells. **b)** Morphological and myelination characteristics of LII-III pyramidal cells.
